# Genetic Variations of *DAOA* (rs947267 and rs3918342) and *COMT* Genes (rs165599 and rs4680) in Schizophrenia and Bipolar I Disorder

**DOI:** 10.32598/bcn.9.6.429

**Published:** 2018-11-01

**Authors:** Leila Ahmadi, Seyed Reza Kazemi Nezhad, Parisima Behbahani, Nilofar Khajeddin, Mehdi Pourmehdi-Boroujeni

**Affiliations:** 1. Department of Genetics, Faculty of Sciences, Shahid Chamran University of Ahvaz, Ahvaz, Iran.; 2. Department of Psychiatry, School of Medicine, Ahvaz Jundishapur University of Medical Sciences, Ahvaz, Iran.; 3. Department of Food Hygiene, Faculty of Veterinary Medicine, Shahid Chamran University of Ahvaz, Ahvaz, Iran.

**Keywords:** Catechol-O-methyltransferase, D-amino acid oxidase activator, Genetics, Schizophrenic disorders, Bipolar disorder

## Abstract

**Introduction:**

Genetic and environmental factors are involved in the incidence of schizophrenia and bipolar disorder. Many reports confirm that several common genes are connected with these two psychotic disorders. Several neurotransmitters may be involved in the molecular mechanisms of schizophrenia and bipolar disorder. We aimed to estimate the role of two talent genes: *DAOA* in neurotransmission of glutamate and *COMT* in neurotransmission of dopamine to guide the treatment of schizophrenia and bipolar disorder.

**Methods:**

Blood samples (n=100 for schizophrenia, n=100 for bipolar I disorder and n=127 for case control) were collected from individuals unrelated in the southwest of Iran. The SNPs (rs947267 and rs3918342 for *DAOA* gene/rs165599 and rs4680 for *COMT* gene) were genotyped using the PCR-RFLP method. Our finding was studied by logistic regression and Mantel-Haenszel Chi-square tests.

**Results:**

We observed an association in rs3918342, rs165599 and rs4680 single nucleotide polymorphisms and schizophrenia and bipolar I disorder. In addition, our data demonstrated that the rs947267 was related to bipolar I disorder but there was no association between this SNP and schizophrenia.

**Conclusion:**

In conclusion, this result supports the hypothesis that variations in *DAOA* and *COMT* genes may play a role in schizophrenia and bipolar disorder.

## Highlights

- This is the first study that examines the association of the rs1656688 and rs4680 of *COMT* gene and rs947267 and rs3918342 of *DAOA* gene in Iranian population.
- The present study aimed to extensively evaluate the contribution of *DAOA* and *COMT* genes in susceptibility to schizophrenia and bipolar I disorder.
- Our data provided further evidence that the *DAOA* locus or *COMT* locus may contribute in the pathophysiology of psychotic disorders.

## Plain Language Summary

Schizophrenia is a serious mental illness that interferes with the person’s ability to think clearly, manage emotions, make decisions and relate to others. Bipolar disorder is a brain disorder that causes unusual shifts in mood, energy, activity levels, and the ability to carry out day-to-day tasks. Studies have shown that these ailments are affected by genetic and environmental factors. *DAOA* and *COMT* genes are two potential candidates for involvement in schizophrenia and bipolar disorder molecular mechanisms. Therefore, this study aims to evaluate the role of these genes in order to improve the present treatments for these illnesses. For this purpose, the association of four desired positions with schizophrenia and bipolar disorder was investigated. The results showed that three of these positions were associated with both diseases and one position was associated with bipolar I. Thus, there are possibilities for *DAOA* and *COMT* genes variations to be involved in schizophrenia and bipolar I disorders.

## 1. Introduction

Schizophrenia (SCZ) is a serious mental disorder that approximately 1% of the world’s population suffer from it (Yue et al., 2007). SCZ is characterized by delusions, hallucinations, thought disorders and cognitive deficits (Rees, O’Donovan, & Owen, 2015). Bipolar Disorder (BD) presents with diverse clinical manifestations. It is characterized by episodes of mania or hypomania. It usually categorized into Bipolar I Disorder (BID) and Bipolar II Disorder (BIID). BID is mainly characterized by depressive and manic symptoms. Moreover, patients may experience psychotic features like delusion and hallucination. These patients usually have the indication for residential treatment (Holtzman, Lolich, Ketter, & Vázquez, 2015).

Family, twin and adoption studies uniquely illustrate the role of genetic agents in transition of SCZ and BD (Boks et al., 2007). The heritability estimates of SCZ and BD are 80% (Aleman, Kahn, & Selten, 2003) and 80%–90%, respectively (Leahy, 2007). Several neurotransmitters such as glutamate (Nasirizade, Mostofi, & Shahbazi, 2016), dopamine, GABA (Rahmanzade et al., 2017) (Dehghani, & Shahbazi, 2016), and serotonin engage in the molecular mechanisms of SCZ and BD (Austin, 2005). Dopamine is an inhibitory neurotransmitter and glutamate is an excitatory neurotransmitter, involved in a variety of neural processes (Goff & Coyle, 2001). The dopamine and glutamate hypotheses are leading theories of the pathoaetiology of SCZ (Howes, McCutcheon, & Stone, 2015).

Neurobiological linkage and association studies suggest that the susceptibility genes in SCZ and BD can be divided into 2 main classes. The first class genes (*DAOA*, NRG1, DISC1, dysbindin and GRM3) affect the NMDA glutamate receptor. The second class genes which include *COMT*, *DRD2* and *PPP1R1B* are involved in dopamine metabolism and signaling (Harrison & Weinberger, 2005). *DAOA* gene (13q34) and *COMT* gene (22q11) are not only associated with psychotic disorders, but also play a key role in glutamatergic and dopaminergic neurotransmissions (Ross, Margolis, Reading, Pletnikov, & Coyle, 2006).

Chumakov et al. (2002) identified *DAOA* gene (D-amino acid oxidase activator). The *DAOA* protein functions as an activator of DAAO (D-amino-acid oxidase). DAAO gene (12q24) oxidizes D-serine which is a potent activator of N-Methyl-D-Aspartate (NMDA). NMDA receptor is a postsynaptic Glutamate Receptor (GluRs) in the human brain (Maderia, Freitas, Vargas-Lopes, Wolosker, & Panizzutti, 2008). Glutamate is an excitatory neurotransmitter, involved in a variety of neural activities including synaptic flexibility, neuronal development, and neuronal toxicity (Goff & Coyle, 2001). Normal glutamatergic neurotransmission involves enzymes, pre- and post-synaptic neurons, glial cells, glutamate receptors and transporters. Disruption in any of the items may interrupt normal glutamatergic neurotransmission (Meador-Woodruff & Healy, 2000).

*DAOA* and DAAO genes interact in the NMDA receptor regulation pathway in SCZ and BD. “Glutamate hypothesis” derived from NMDA antagonists like Phencyclidine (PCP) and ketamine can cause psychotic and cognitive abnormalities in SCZ (Ross et al., 2006). Likewise, “dopamine hypothesis” originated from the identification of D2 receptor blockage. The mechanism of action is similar in D2 receptor blockage and antipsychotics (Dashti, Aboutaleb, & Shahbazi, 2013). Catechol-O-methyltransferase (*COMT*) is a unique enzyme for decomposing a number of bioactive molecules like dopamine. This enzyme is encoded by the *COMT* gene (Lotta et al., 1995).

*COMT* gene is located in 22q11, a region that is a source of confusion in many linkage analysis (Lewis et al., 2003). Deletions in 22q11 can also lead to the velocardiofacial syndrome through an increased risk of psychopathy (Karayiorgou et al., 1995). Not all studies have supported the *DAOA* and *COMT* genes association with SCZ and BD (Liu et al. 2006; Shi, Badner, Gershon, & Liu, 2008; Tan et al., 2014; Jagannath et al. 2017); however, genome wide association studies of *DAOA* and *COMT* genes with SCZ and BD are available (Shifman et al., 2002; Glatt, Faraone, & Tsuang, 2003; Shifman et al., 2004; Sacchetti et al., 2013; Gatt, Burton, Williams, & Schofield, 2015); Chu et al., 2017; Jagannath, Gerstenberg Correll, Walitza, & Grünblatt, 2018). We postulated the genetic variation of *DAOA* gene in glutamate neurotransmission and *COMT* gene in dopamine neuro-transmission, to facilitate the treatment of SCZ and BD. We also assessed the impact of those on susceptibility for SCZ and BD.

## 2. Methods

### 2.1. Sampling

To collect the study samples, we used General Health Questionnaire-28 (GHQ-28) (Lobo, Perez-Echeverria, & Artal, 1986) and Diagnostic and Statistical Manual of Mental Disorders (DSM-IV). The patients were attended by at least 2 psychiatrists since admission. All the patients were treated with mood stabilizers or antipsychotics, during the study period. The control group consisted of 127 non-relative individuals who were screened in 2 steps.

First, they were asked whether their first and second degree relatives have a history of at least one of the following problems: taking mental health medications, referral to a psychiatrist or psychologist, psychiatric hospitalization, substance abuse or dependence, and suicide attempts. Second, the screening was completed by General Health Questionnaire. There was no remarkable diversity in gender distribution among the cases and controls (55%, 67% and 47% of the controls, SCZ patients and BD patients were males, respectively). The controls, BD patients and SCZ patients had Mean±SD age of 37.6±9.6, 34.4±11.2 and 36.9±10.2 years, respectively.

### 2.2. DNA extraction

Blood samples (n=127 for the controls, n=100 for schizophrenia and n=100 for BID) were collected from non-relative individuals in the southwest of Iran. The total genomic DNA was extracted from the leukocytes using Diatom DNA Prep extraction kit (Gene Fanavaran, Iran), based on the structures. A spectrophotometer was applied to determine the density of genomic DNA.

### 2.3. SNP genotyping and statistical analysis

We selected Single Nucleotide Polymorphisms (SNPs) from the public SNP database, dbSNP (http://www.ncbi.nlm.nih.gov), as well as the published findings (Table 1). We chose the markers (rs947267, rs3918342) for *DAOA* gene and (rs165599, rs4680) for *COMT* gene, because these genes are associated with SCZ and BD. Many studies have recommended that gene polymorphisms are associated with gene expression. The rs4680 is located in exonic region. Exonic SNPs directly impact the characteristics of proteins, while SNPs within untranslated region and introns affect the expression and splicing of mRNA.

**Table 1:** Description of genotyped markers

**Genes**	**SNPs**	**Chr Position (bp)**	**Alleles**
*DAOA* (13q34)	rs947267	105487313	A/C
	rs3918342	105533400	C/T
*COMT* (22q11)	rs165599	19969258	A/G
	rs4680	19963748	A/G

The rs3918342 is located upstream of 5′UTR and the rs165599 is located on 3′UTR. The sequences of the UTRs (untranslated regions) of mRNAs play significant roles in post-transcriptional management. However, it is unclear whether change in UTR length can significantly affect the regulation of gene expression (Lin & Li, 2012). The rs947267 is located in the intronic region. Intronic region mutations induce abnormal splicing (e.g. cryptic splice sites or exon skipping) that is obviously different from normal alternative splicing (Cooper, 2010).

The DNA samples were used to genotyping by PCRRFLP methods. Polymerase Chain Reaction (PCR) is a technique used in molecular biology to amplify a single copy or a few copies of a segment of DNA for producing thousands to millions copies of a special DNA sequence. As demonstrated in Table 2, the samples were amplified by 2 primer pairs. Primers were designed using the Primer3 software or NCBI Primer-Blast (http://www.ncbi.nlm.nih.gov/tools/primer-blast), with the parameters to create a product set.

**Table 2:** Primer sequences of the rs947267, rs3918342, rs165599 and rs4680 SNPs

Genes	SNPs	Alleles	Primers
*DAOA*	rs947267	A/C	Forward: 5′-GGGAAAAGGTATCAGGGAGAG-3′ Reverse: 5′-TTGCACACGAACCAAATCAG-3′
	rs3918342	C/T	Forward: 5′-GGAAACCAGAAGGTGAAA-3′ Reverse: 5′-GAATCAGAAAGGAAAAGTGT-3′
*COMT*	rs165599	A/G	Forward: 5′-CACAGTGGTGCAGAGGTCAG-3′ Reverse: 5′-CTGGCTGACTCCTCTTCGTTT-3′
	rs4680	A/G	Forward: 5′-TCATCACCATCGAGATCAACC-3′ Reverse: 5′-CCCTTTTTCCAGGTCTGACA-3′

In Restriction Fragment Length Polymorphism (RFLP) analysis, the DNA sample is broken into pieces (and digested) by restriction enzymes and the resulting restriction fragments are separated according to their lengths by gel electrophoresis. RFLP analysis can be used as a form of genetic testing to observe whether an individual carries a mutant gene for a disease that runs in his or her family.

**Figure 1:**
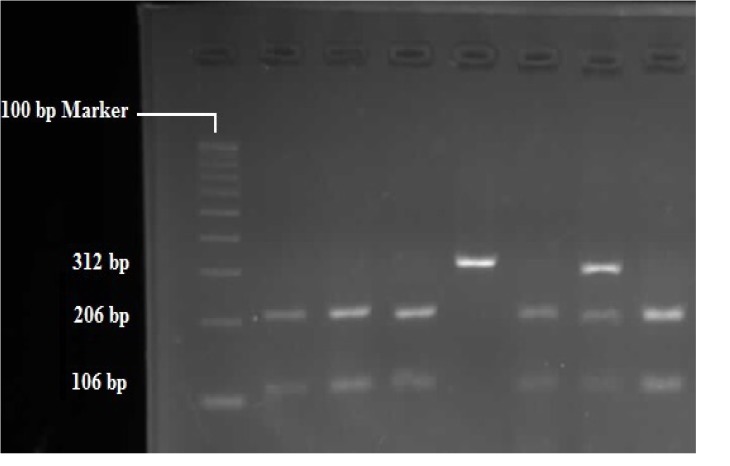
RFLP of rs947267 by HaeIII restriction enzyme: AA (312 bp), CC (206 bp/106 bp), AC (312 bp/206 bp/106 bp)

**Figure 2:**
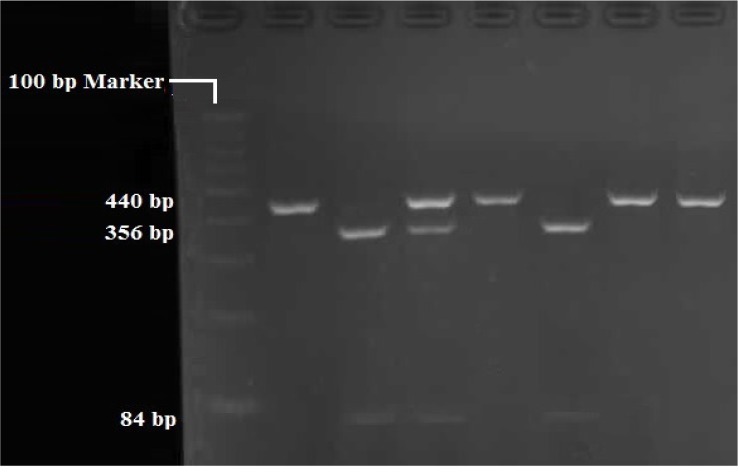
RFLP of rs3918342 by BsaAI restriction enzyme: TT (440 bp), CC (356 bp/84 bp), TC (440bp/356bp/84 bp)

**Figure 3:**
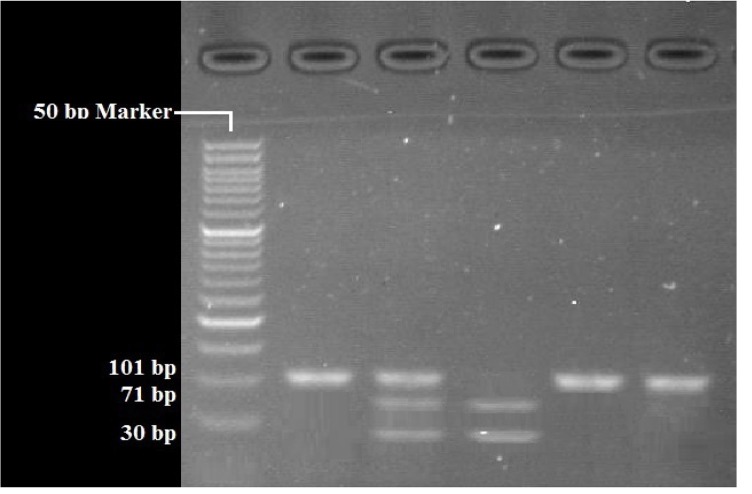
RFLP of rs165599 by MspI restriction enzyme: AA (101 bp), GG (71 bp/30 bp), AG (101bp/71bp/30 bp)

**Figure 4:**
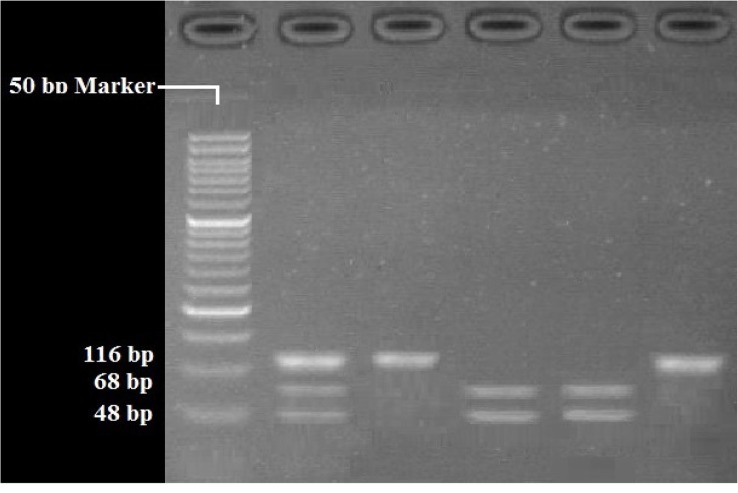
RFLP of rs4680 by Hin1II restriction enzyme: GG (116 bp), AA (68 bp/48 bp), AG (116bp/686bp/48 bp)

RFLP analysis was performed to determine genotypes of 4 polymorphisms; rs947267 by HaeIII restriction enzyme (Figure 1), rs3918342 by BsaAI restriction enzyme (Figure 2), rs165599 by MspI restriction enzyme (Figure 3), and rs4680 by Hin1II restriction enzyme (Figure 4). In addition, the data were confirmed by sequencing assay. DNA sequencing is the process of determining the http://bcn.iums.ac.ir/ precise order of nucleotides within a DNA molecule. Information were studied by logistic regression and Mantel-Haenszel Chi-square tests. “Hardy Weinberg equilibrium” was estimated using Chi-square test.

## 3. Results

The present study aimed to extensively evaluate the contribution of *DAOA* and *COMT* genes in susceptibility to SCZ and BID. Genotypic distribution of the SNPs in the case and control groups are listed in Table 3. Moreover, the allele frequency of these SNPs in the case and control groups are listed in Table 4. The obtained data were found in Hardy Weinberg equilibrium. The allele frequency of SNPs was studied by logistic regression and Mantel-Haenszel Chi-square tests. The significance level was set at P<0.05. As per Table 4, the results of P values revealed a statistically significant association between SNPs rs3918342 (P=0.001), rs165599 (P<0.001) and rs4680 (P<0.001), with SCZ. In addition, there was a significant association between SNPs rs3918342 (P<0.001), rs165599 (P<0.001) and rs4680 (P=0.02) with BID.

**Table 3:** Genotypic distribution of the SNPs in the case and control groups

**SNPs**	**Genotypic Distribution**	**%**		

		**Schizophrenia**	**Bipolar I Disorder**	**Controls**
rs947267	AA	39	41	30.7
	CC	12	2	22.85
	AC	49	57	46.45
rs3918342	TT	46	23	52.755
	CC	17	22	4.725
	TC	37	55	42.52
rs165599	AA	41	60	16.5
	GG	8	3	44.9
	AG	51	37	38.6
rs4680	AA	94	67	74
	GG	0	10	1
	AG	6	23	25

**Table 4:** Allelic frequency and P values of the SNPs in the case and control groups

**SNPs**	**Case and Control**	**Allelic Frequency**		**P**
rs947267	Schizophrenia	C	0.365	0.09
		A	0.635	
	BID	C	0.305	<0.001
		A	0.695	
	Controls	C	0.54	
		A	0.46	
rs3918342	Schizophrenia	C	0.355	0.001
		T	0.645	
	BID	C	0.495	<0.001
		T	0.505	
	Controls	C	0.26	
		T	0.74	
rs165599	Schizophrenia	A	0.665	<0.001
		G	0.335	
	BID	A	0.785	<0.001
		G	0.215	
	Controls	A	0.358	
		G	0.642	
rs4680	Schizophrenia	A	0.97	<0.001
		G	0.03	
	BID	A	0.785	0.02
		G	0.215	
	Controls	A	0.865	
		G	0.135	

Our data demonstrated that rs947267 (P<0.001) was significantly related with BID; however, there was not any association between this SNP (P=0.09) and SCZ. Our data provided further evidence that the *DAOA* locus or *COMT* locus may play roles in the pathophysiology of psychotic disorders. Although no direct link has been revealed between genetic polymorphism in these genes and NMDA receptor function, the present results support previous reports implicating the *DAOA* as susceptible genes for psychotic disorders. Further investigation is warranted to determine the functional variation underlying these results and relate this to the pathophysiology of psychotic disorders.

## 4. Discussion

The genetics of SCZ and BD seem complicated and without a specific heritability. A multi-locus model has been suggested to clarify the pattern of heritability in this complex disorder. This model indicates that a composition of various genetic agents evolve in these disorders (Risch, 1990). Hence, more than one locus affects the development of SCZ and BD. According to the studies on glutamatergic pathway in Iran, *PRODH* gene (Rahman zadeh, Mohammadi, karimipour, Heidari keshel, & Omidinia, 2012), *DTNBP1* gene (Galehdari, Ajam, & Pooryasin, 2010), *GRIN1* gene (Galehdari, 2009), dysbindin gene (Alizadeh, et al., 2012), and *NRG1* gene (Shariati, Behmanesh, & Galehdari, 2011) are associated with SCZ.

Dopaminergic pathway research studies in Iran reported that DISC1 gene, is not associated with schizophrenia (Foroughmand, et al., 2010). However, MAOA gene is correlated with BD (Amirabadi, et al., 2015). In this study, the key role of 2 candidate genes; *DAOA* gene (13q34) and *COMT* gene (22q11) were significant.

Our findings were compared with other studies. In a meta-analysis, 13 genetic variants revealed genetic overlap between 2 or more affective disorders. *DAOA* (rs3918342), *COMT* (Val158Met), *DRD4* 48-bp, *DAT1* 40-bp, *SLC6A4* 5-HTTLPR, *APOE e4*, ACE Ins/Del, *BDNF* (Val66Met), *HTR1A* C1019G, *MTHR* C677T, MTHR A1298C, *TPH1* 218A/C and *SLC6A4* (VNTR) are demonstrating evidence for pleiotropy in affective disorders (Gatt et al., 2015). Hukic proposed an interaction between *DAOA* and *COMT* genes. In addition, SNPs in this genes were associated with cognitive dys-function in bipolar patients (Hukic, 2016).

A study on an Italian population presented some evidence for the association between NMDA-receptor-mediated signaling genes, DAO, PPP3CC, *DAOA* and DTNBP1 with SCZ (Sacchetti et al., 2013). Deficiency in the glutamatergic system is involved in the pathophysiology of both SCZ and BD (Tsai & Coyle, 2002). The A allele of rs947267 was associated with BID in our study. A follow-up subgroup analysis suggested the genetic polymorphisms of rs947267 in the *DAOA* gene were not a statistically significant increased risk for SCZ and BD, among the Asian and Caucasian population (Tan et al., 2014).

A meta-analysis composed of 18 correlational articles suggested no correlation between rs947267 and BD, while a remarkable association between rs947267 and BD has been reported in Iran (Shi et al., 2008). Moreover, the association between rs947267 and both SCZ and BD is quite different in the Southwest of Iran, compared to the Asian population. This meta-analysis suggested a correlation between rs947267 and SCZ. Such association has not been observed in Iran. In addition, the T allele of rs3918342 was associated with schizophrenia and BID, in our study. This is the same allele associated with the above-mentioned disorders, in the study of Chumakov et al. (2002).

The meta-analysis on *DAOA* studies reported no association between rs3918342 and SCZ (Shi et al., 2008). Moreover, the genetic polymorphisms of rs3918342 in the *DAOA* gene revealed no statistically significant increased risk of SCZ and BD, in a follow-up subgroup analysis on Caucasian and Asian population (Tan et al., 2014). However, the SNP rs3918342 of the *DAOA* gene showed significant association with SCZ in the Taiwanese population (Chu et al., 2017). Likewise, a remarkable association has been observed between rs3918342 and SCZ and BD in the United Kingdom (Bass et al., 2009).

With regards to the *COMT* gene, the A allele of rs165599 and rs4680 single nucleotide polymorphisms were associated with SCZ and BID, in our study. A study on Ashkenazi Jewish patients highlighted the significance of association between *COMT* gene and SCZ (Shifman et al., 2002). Shifman et al. (2004) also reported a positive correlation between rs165599 and BD. Many researchers have studied the rs4680 polymorphism of *COMT* gene. The association of this variant with SCZ is complex and might be influenced by genetic substructure of human populations (Glatt et al., 2003).

Shifman et al. (2004) observed the association between schizophrenia and rs4680. However, Lajin, Alachkar, Hamzeh, Michati and Alhaj (2011) did not find any association between rs4680 and SCZ. In addition, Shifman et al. (2004) reported no association between rs4680 and BD. However, Mynett-Johnson demonstrated an association between rs4680 and BD (Mynett-Johnson Murphy, Claffey, Shields, & McKeon, 1998).

Obviously, our study fails to prove or reject the complexity of glutamate and dopamine neurotransmission. However, it presents confirmation for the association between such neurotransmissions and SCZ and BD in the patients living in the Southwest Iran. Available treatments for psychotic disorders have had partial success, because most of the work on psychotic disorders was only focused on dopamine for approximately 40 years. While glutamate is the most abundant excitatory neurotransmitter in the nervous system and plays a key role in most aspects of normal brain functions, including cognition, memory and learning. Moreover, revealing the association between SCZ and BD with *DAOA* and *COMT* genes recreate the glutamate and dopamine hypothesis.

Genetic linkage analysis has identified numerous overlapping regions in these disorders, including chromosome 6p, 13q, 18q and 22q (Badner & Gershon, 2002). In addition, based on many genetic observations, first or second degree relatives of schizophrenia or bipolar disorder patients are at high risk for these 2 disorders (Arajärvi et al., 2006). SCZ and BD present overlapping symptoms, despite separate and exclusive diagnostic criteria, defined for each. Hence, the etiologic segregation of these disorders into homogenous subtypes is currently under debate.

Our findings suggest a positive correlation between *DAOA* and *COMT* genes with SCZ and BD. Our results may provide more validation for the existence of genetic overlap in the common genes of schizophrenia and bipolar disorder.

## Ethical Considerations

### Compliance with ethical guidelines

All procedures performed in studies were in accordance with the ethical standards of the institutional research committee and comparable ethical standards.
